# Cannabis use is not associated with altered levels of physical activity: evidence from the repeated cross-sectional Belgian Health Interview Survey

**DOI:** 10.1186/s42238-025-00278-8

**Published:** 2025-04-25

**Authors:** Brent Vernaillen, Brecht Devleesschauwer, Stijn Vansteelandt, Lydia Gisle, Sabine Drieskens, Elena Damian

**Affiliations:** 1 Department of Epidemiology and Public Health, Sciensano, Brussels, Belgium; 2 Department of Applied Mathematics, Computer Science and Statistics, Ghent University, Ghent, Belgium; 3 Department of Translational Physiology, Infectiology and Public Health, Ghent University, Merelbeke, Belgium; 4 Current address: Department of Experimental Psychology, Ghent University, H. Dunantlaan 2, Ghent, 9000 Belgium

**Keywords:** Cannabis, Physical activity, Belgium, Health Interview Survey, Propensity, Complex survey design, Exercise, Propensity Scores

## Abstract

**Background:**

Several studies have suggested a positive effect of occasional cannabis consumption on the frequency of leisure-time physical activity, possibly due to more motivation before, more enjoyment during, and better recovery after engaging in leisure-time physical exercise. While such an effect would contradict the stereotypical image of lower physical activity levels in cannabis users as compared to non-users, evidence has been mixed at best. The current study investigated this proposed association in a representative sample of the Belgian population.

**Methods:**

Data from four waves of the Belgian Health Interview Survey (HIS; repeated cross-sectional survey; 2001 – 2018) were used in a regression and propensity matching analysis to examine the association between past-month cannabis use and physical activity levels, while controlling for potentially confounding variables. A total of *n* = 19,936 individuals (48.9% female) aged 15–64 years were included in the analysis. We modelled physical activity in function of past-month cannabis use while adjusting for potential confounders.

**Results:**

Both the regression analysis and the propensity-matching analysis revealed no evidence in favor of a positive effect of past-month cannabis use on physical activity level (estimated OR = 0.97, 95% CI = [0.74, 1.28] and estimated RR = 0.90, 95% CI = [0.70; 1.16] respectively). Descriptive analyses of baseline characteristics suggested some clear differences between users and non-users that were in line with previous studies.

**Conclusions:**

There was no evidence suggesting that past-month cannabis users have better or worse physical activity levels compared to non-users in the Belgian population aged 15–64 years.

**Supplementary Information:**

The online version contains supplementary material available at 10.1186/s42238-025-00278-8.

## Introduction

Over the past few years the subject of cannabis use and its effects on physical and mental health has become a very popular topic, especially in the debate on legalization of recreational cannabis use in many countries (The Lancet Regional Health- Europe 2021). According to the United Nations world drug report (European Monitoring Centre for Drugs and Drug Addiction 2022), cannabis is the most popularly used illicit drug in the general population, with an estimated 27.3% of all people aged between 15 and 64 having used cannabis at least once in their lifetime. Furthermore, the report revealed an increasing trend in the average potency of the used substances (measured as the concentration in delta- 9-tetrahydrocannabinol, %THC, the most important psychoactive component of cannabis) between 2010 and 2020.

In Belgium, where both possession and consumption of recreational cannabis is illegal, but decriminalized, the proportion of adults aged 15 to 64 years who used cannabis at least once in their lifetime increased from 15% in 2013 to 22.6% in 2018 (Gisle and Drieskens 2018). This increasing trend was also observed in past-year use, with an increase from around 5% in 2004–2013 to around 7% in 2018, and past-month cannabis use with around 3% in 2001–2013 to 4.3% in 2018 (Gisle and Drieskens 2018). This relatively high and increasing prevalence, both worldwide and in Belgium, together with increasing potency highlight the importance of extensive research on the health effects of cannabis.

Apart from the known adverse effects (mainly related to the psychoactive THC component) on the cardiovascular system (Ghosh and Naderi 2019; Page et. al 2020), the respiratory system (Gates et al. 2014; Winhusen et al. 2019), Body Mass Index (BMI; (Fearby et al. 2022), and mental health (Moore et al. 2007), some studies point towards potential health benefits of recreational cannabis consumption (predominantly attributed to the cannabidiol component, CBD; (YorkWilliams 2019; Gillman et. al 2015; Ong et. al 2021; Gibson et al. 2023; Korn et al. 2018). Particularly, an increasing number of studies suggest that, on average, people who use cannabis (referred to as users) tend to engage more in physical activity compared to people who do not use cannabis (referred to as non-users, (YorkWilliams 2019; Gillman et. al 2015; Ong et. al 2021; Gibson et al. 2023; Korn et al. 2018).

YorkWilliams et al. (2019) conducted an online survey in the United States of America to investigate the link between cannabis use and physical exercise among adults living in the states where cannabis is fully legalized. The authors found that participants using cannabis in close proximity to exercise performed on average 30.2 min (95% Confidence Interval (CI) = [2.4; 58.0]) more anaerobic exercise per week compared to participants who did not. Moreover, the majority of users reported greater enjoyment while exercising and better recovery afterwards. Though this study did not include a non-using control group and collected data using self-reports, the results nonetheless highlight potential benefits of occasional cannabis consumption on physical activity. Echoing these findings, Smith et al. (Smith et al. 2021) observed that ever having used cannabis was associated with higher odds of physical activity, with a similar pattern also observed by Ong et al. (2021).

Three main components that may underlie this potential association are commonly suggested: heightened motivation to exercise (YorkWilliams 2019; Gibson 2024), increased enjoyment during exercise (Dietrich 2004), and faster recovery after exercise (Schubert et. al 2022; Kozela et al. 2013; Nagarkatti et al. 2009). The first two components have often been related to the “runner’s high” (i.e., feeling euphoric and relaxed after prolonged anaerobic activity). Related to faster recovery after exercise, the anti-inflammatory effects of cannabis (both THC and CBD) are assumed to play a crucial role. (Gillman et. al 2015; Kozela et al. 2013; Nagarkatti et al. 2009).

Gibson and Bryan (Gibson 2024) illustrated the acute effect of cannabis consumption on subjective experiences during exercise using a within-subjects crossover design. In this study, experiences such as negative and positive affect, exercise enjoyment and the runner’s high were measured during physical activity with and without prior cannabis consumption. Results revealed that positive- and negative affect while engaging in physical exercise were respectively elevated and decreased in the cannabis condition. Additionally, lower levels of physical pain and a stronger runner’s high were also observed when using cannabis.

Importantly, there is some evidence suggesting that cannabis (and particularly THC) has important interactions with both the human endocannabinoid system (Hanney 2022) and neural reward processing system (Bloomfield et al. 2016), which could explain the runner’s high, increased enjoyment, and elevated motivation associated with cannabis use (Gillman et. al 2015).

So far, research investigating the association between cannabis use and physical activity has been somewhat limited, and has led to inconclusive results. On the one hand, studies suggest that cannabis either has no effect (Gibson et al. 2023; YorkWilliams et al. 2020), or even a detrimental effect on exercise performance (Pesta et al. 2013; Kennedy 2017). On the other hand, there are the previously mentioned studies that point towards a potential positive association between occasional cannabis consumption and activity (YorkWilliams 2019; Gillman et. al 2015; Ong et. al 2021; Gibson et al. 2023; Korn et al. 2018). Taken together, evidence regarding the aforementioned association is mixed at best, warranting the need for further investigation. This study aimed to further explore the association between past-month cannabis use and physical activity by analyzing data from a large national health survey (the Belgian Health Interview Survey, HIS, 2001–2018; (Gisle and Drieskens 2018; Demarest 2018; Demarest et al. 2013; Sciesano n.d.). To this end, we conducted a survey-weighted logistic regression with propensity score adjustment as well as a propensity score matched analysis. To the best of our knowledge, this is the first investigation of this association in a representative sample of the general Belgian population.

## Methods

### Dataset

The data used in this study were part of the HIS dataset, resulting from a repeated cross-sectional survey conducted among the Belgian population (Gisle and Drieskens 2018; Demarest 2018; Demarest et al. 2013; Sciesano n.d.) by Sciensano every four to five years. This survey gathers data on a wide range of health- and lifestyle-related topics from a large, representative sample of the Belgian population. Currently, data from six waves are available (years 1997, 2001, 2004, 2008, 2013 and 2018). The current study used data from wave two (2001) up to, and including wave six (2018) as certain variables of interest were not (or only partly) recorded in the first wave. The final sample following pre-processing (see Supplementary Material 1) consisted of 19,936 individuals aged 15–64 years. Data were weighted using the post-stratification weights of the HIS dataset to represent the target Belgian population.

### Measures

#### Dependent variable

The dependent variable was ‘Leisure-time physical activity’ consisting of two response categories (‘mainly sedentary activities’ and ‘light/intensive physical activities’) in order to allow for binary logistic regression (see Supplementary Material 1 for more information on the creation of this variable).

#### Independent variable

The main predictor variable of interest, past-month cannabis use, was measured by a binary variable indicating cannabis use in the past 30 days. With this variable, we aimed to better target current users as opposed to past-year or even lifetime users (see Discussion).

#### Covariates

Covariates were selected based on the literature suggesting a potential association with either the dependent or the independent variable. By including these covariates (see Supplementary material 1), we aimed to minimize potential confounding of the relationship between past-month cannabis use and physical activity. Selected covariates were age (Jeffers et al. 2021; Trost et al. 2002; Mota and Esculcas 2002), education (Jeffers et al. 2021; Lynskey and Hall 2000; Macleod et al. 2004; Shaw and Spokane 2008), income (Jeffers et al. 2021; Ford et al. 1991), sex (Jeffers et al. 2021; Mota and Esculcas 2002; Cranford et al. 2009; Carliner et al. 2017), the Global Activity Limitation Indicator (GALI, (Oyen et al. 2006), degree of urbanization (Reis et al. 2004), and depression and anxiety (Macleod et al. 2004; Lev-Ran et al. 2014; Degenhardt et al. 2003; Crippa et al. 2009; Hayatbakhsh et al. 2007). Additionally, we included the variables Year of the survey and Province of residence to further control for differences in time and geographical location.

### Statistical analyses

#### Regression analysis

As a result of the stratified multi-stage clustered sampling design of the HIS, some methodological aspects need to be taken into consideration (see Supplementary Material 1 for the exact motivation). Both unequal selection probabilities for individuals in the target population, and the clustered structure (e.g., people nested within households) were taken into account by using the surveylogistic procedure in SAS version 9.4 (SAS Institute Inc. 2016). Additionally, we used estimated propensity scores as predictors (i.e., the estimated probability of using cannabis in the last 30 days given the baseline covariates, estimated here using a logistic regression model that takes into account the complex survey design; see and Supplementary Material 1). This also allowed us to evaluate differences in baseline covariates between users and non-users. The final regression model contained physical activity as the outcome and all covariates together with past-month cannabis use and the estimated propensity score as predictors. Model-building proceeded via the method of double-variable selection. Sampling weights representing how many individuals in the target population were represented by each individual in the sample were used (see Supplementary Material 1).

#### Propensity score matching analysis

A potential drawback of the regression framework is that the model coefficients have a subtle interpretation: these capture the natural logarithm of the estimated odds ratio comparing two levels of a covariate (e.g. users vs non-users). An alternative analysis yielding more easily interpretable estimates, and that is less vulnerable to extrapolation, uses propensity scores to match comparable non-users to past-month cannabis users, delivering estimates of the average effect of cannabis in the user population (see Supplementary Material 1). This effect expresses how much the percentage of physically active users would change in the users if they had not used cannabis in the last 30 days. Analysis of the matched dataset was carried out with the surveyfreq procedure in SAS software (SAS Institute Inc. 2016).

#### Sensitivity analysis

We conducted a sensitivity analysis to evaluate the robustness of our results against minor changes to either the regression model or the propensity estimation model. To this end, we ran the same regression analysis with all two-way interactions between covariates included, both for estimating the propensities and in the final regression model, as well as analyses including a quadratic effect of age in the propensity score model and the final regression model (see Supplementary Material 2).

## Results

### Descriptives

The current section discusses the weighted and unweighted descriptive summary statistics of the 19,936 participants included in the statistical analyses (i.e., the complete cases remaining after 42,959 incomplete/inapplicable observations were omitted following pre-processing, see Supplementary Material 1).

#### Description of the sample (unweighted summary statistics)

**Fig. 1:**
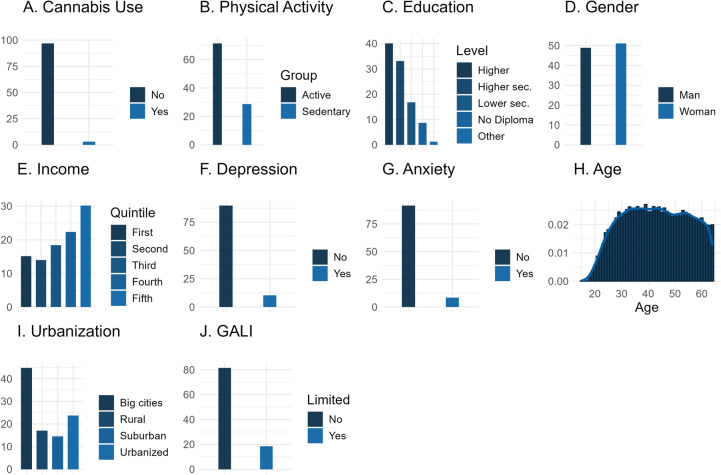
Unweighted descriptive summary statistics for all variables included in the analysis. Legend: The current plots provide insight into the variable distributions in study sample based on pre-processed, unweighted data Y-axes represent percentages, except for panel G (Age) where it represents a density

Past-month cannabis use was relatively rare in the sample with only 3.1% of respondents (*n* = 618) answering positively to this question (Fig. 1A). 71.3% of respondents (*n* = 14,223) were in the category of light/intensive activity (Fig. 1B). An overview of all variables can be found in Table 1.

**Table 1:** Descriptive statistics of categorical variables, univariate and conditional on cannabis use or physical activity

**Variable**	**Total**			**Cannabis**						** Physical Activity**					
				**Yes**			**No**			**Active**			**Sedentary**		
	**n**	**% (U)**	**% (W)**	**n**	**% (U)**	**% (W)**	**n**	**% (U)**	**% (W)**	**n**	**% (U)**	**% (W)**	**n**	**% (U)**	**% (W)**
Cannabis															
Yes	618	3.1	2.8							431	3.0	2.8	187	3.3	3.0
No	19318	96.9	97.2							13792	97.0	97.2	5526	96.7	97.0
Physical															
Active	14223	71.3	73.1	431	69.7	71.5	13792	71.4	73.1						
Sedentary	5713	28.7	26.9	187	30.3	28.5	5526	28.6	26.9						
Education															
No Diploma	1737	8.7	8.0	29	4.7	3.8	1708	8.8	8.1	939	6.6	6.2	798	14.0	13.0
Lower sec.	3351	16.8	16.1	99	16.0	15.3	3252	16.8	16.1	2132	15.0	14.4	1219	21.3	20.7
Higher sec.	6601	33.1	35.4	232	37.5	42.9	6369	33.0	35.2	4586	32.2	34.7	2015	35.3	37.2
Higher	7990	40.1	39.5	246	39.8	36.2	7744	40.1	39.6	6386	44.9	43.8	1604	28.1	27.9
Other	257	1.3	1.0	12	1.9	1.8	245	1.3	1.0	180	1.3	0.9	77	1.3	1.3
Gender															
Man	9754	48.9	50.2	439	71.0	74.6	9315	48.2	49.5	7269	51.1	52.2	2485	43.5	44.8
Woman	10182	51.1	49.8	179	29.0	25.4	10003	51.8	50.5	6954	48.9	47.8	3228	56.5	55.2
Income															
First Quintile	3011	15.1	13.4	129	20.9	16.8	2882	14.9	13.3	1796	12.6	11.3	1215	21.3	19.0
Second Quintile	2790	14.0	13.3	90	14.6	15.3	2700	14.0	13.2	1832	12.9	12.3	958	16.8	16.0
Third Quintile	3665	18.4	18.5	102	16.5	12.6	3563	18.4	18.7	2558	18.0	18.2	1107	19.4	19.3
Fourth Quintile	4457	22.4	24.0	131	21.2	24.9	4326	22.4	24.0	3281	23.1	24.6	1176	20.6	22.3
Fifth Quintile	6013	30.1	30.8	166	26.9	30.3	5847	30.3	30.8	4756	33.4	33.6	1257	22.0	23.4
Depression															
Yes	2051	10.3	9.8	112	18.1	16.1	1939	10.0	9.7	1049	7.4	7.1	1002	17.5	17.4
No	17885	89.7	90.2	506	81.9	83.9	17379	90.0	90.3	13174	92.6	92.9	4711	82.5	82.6
Anxiety															
Yes	1703	8.5	91.7	98	15.9	16.1	1605	8.3	8.1	906	6.4	6.2	797	14.0	14.2
No	18233	91.5	8.3	520	84.1	83.9	17713	91.7	91.9	13317	93.6	93.8	4916	86.0	85.8
Urbanization															
Rural	3389	17.0	14.5	77	12.5	12.9	3312	17.1	14.6	2476	17.4	14.6	913	16.0	14.3
Suburban	2894	14.5	19.2	71	11.5	17.5	2823	14.6	19.3	2123	14.9	19.7	771	13.5	17.8
Urbanized	4724	23.7	28.7	94	15.2	17.0	4630	24.0	29.1	3520	24.7	29.9	1204	21.1	25.6
Big cities	8929	44.8	37.5	376	60.8	52.6	8553	44.3	37.1	6104	42.9	35.8	2825	49.4	42.3
GALI															
Limited	3680	18.5	17.5	110	17.8	17.3	3570	18.5	17.5	2106	14.8	13.9	1574	27.6	27.3
Not Limited	16256	81.5	82.5	508	82.2	82.7	15748	81.5	82.5	12117	85.2	86.1	4139	72.4	72.7

The column ‘Total’ contains absolute (n) and relative (%) frequencies of each category in the final sample. The columns ‘Cannabis’ and ‘Physical activity’ contain frequencies, conditional on the respective column variables. % (U) and %(W) refer to unweighted and weighted percentages respectively

*GALI* Global Activity Limitations Indicator

#### Description of the target population (weighted summary statistics)

**Fig. 2:**
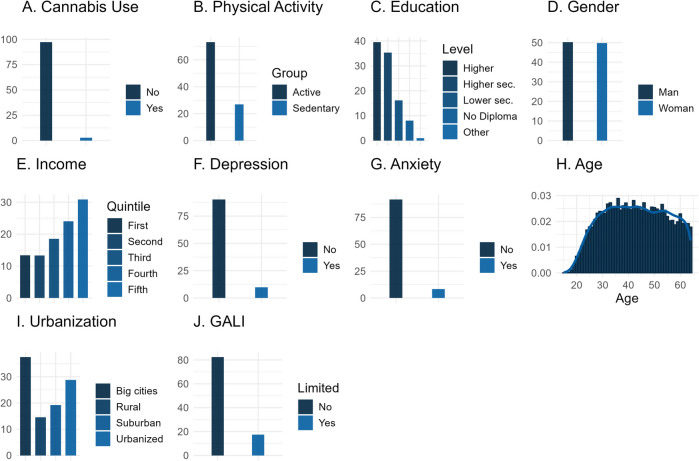
Univariate distributions based on survey-weighted data. Legend: The current plots provide insight into the variable distributions in the target population based on pre-processed, survey-weighted data. Y-axes represent percentages, except for panel G (Age) where it represents a density

In the target population, weighted descriptives revealed that past-month cannabis use was relatively rare with only 2.8% (Fig. 2A). 73.1% of the target population was estimated to be part of the light/intensive activity category (Fig. 2B). An overview of all variables can be found in Table 1.

### Regression results

The regression analysis revealed no statistically significant effect of past-month cannabis use at the *α* = 0.05 significance level when adjusting for the estimated propensity score and covariates (Table 2, see Table 3 for estimated odds ratios and confidence intervals). The odds ratio (OR) of belonging to the light/intensive physical activity group for past-month cannabis users and non-users was estimated to be 0.97 (95% CI = [0.74, 1.28], *p* = 0.84).

**Table 2:** Results of the final outcome regression model (Type III tests)

Variable	F-value	Numerator Df	Denominator Df	*P*-value
Year	5.26	4	13,809	< 0.001^***^
Education	41.19	4	13,809	< 0.001^***^
Cannabis	0.04	1	13,812	0.836
Income	2.41	4	13,809	0.047^*^
Age	17.95	1	13,812	< 0.001^***^
Gender	41.83	1	13,812	< 0.001^***^
Depression	35.53	1	13,812	< 0.001^***^
Anxiety	4.03	1	13,812	0.045^*^
Urbanization	6.03	3	13,810	< 0.001^***^
GALI	93.29	1	13,812	< 0.001^***^
Province	11.24	10	13,803	< 0.001^***^
Year	5.26	4	13,809	< 0.001^***^

*GALI* Global Activity Limitations Indicator

*p* <.05^*^, *p* <.01^**^, *p* <.001^***^

**Table 3:** Estimated odds ratios and 95% confidence intervals for all variables of the final regression model

Variable	Effect	Odds Ratio	95% Confidence Bounds		
			**Lower**	**Upper**	
Year	2001	1.00			
	2004	0.75	0.65		0.86
	2008	0.81	0.71		0.94
	2013	0.81	0.70		0.94
	2018	0.90	0.79		1.03
Education	Higher Education	1.00			
	Higher Secondary	1.59	1.42		1.79
	Lower Secondary	1.95	1.70		2.24
	No Diploma	2.84	2.39		3.37
	Other	1.97	1.30		2.99
Income	First	1.00			
	Second	0.91	0.78		1.07
	Third	0.85	0.73		0.99
	Fourth	0.86	0.73		1.00
	Fifth	0.79	0.68		0.92
Age	Age	0.99	0.99		1.00
Cannabis	No	1.00			
	Yes	0.97	0.74		1.28
Gender	Woman	1.00			
	Man	0.76	0.69		0.82
Depression	No	1.00			
	Yes	1.70	1.43		2.02
Anxiety	No	1.00			
	Yes	1.21	1.01		1.47
Urbanization	Rural	1.00			
	Suburban	1.00	0.84		1.89
	Urbanized	0.97	0.82		1.14
	Big Cities	1.26	1.07		1.47
GALI	Not Limited	1.00			
	Limited	1.77	1.57		1.98
Province	West-Vlaanderen	1.00			
	Antwerpen	0.78	0.63		0.96
	Waals Brabant	1.25	0.94		1.66
	Brussel	1.20	0.99		1.46
	Hainaut	1.71	1.42		2.07
	Limburg	1.06	0.83		1.35
	Liege	1.47	1.19		1.81
	Luxembourg	1.33	1.05		1.67
	Namur	1.19	0.94		1.52
	Oost-Vlaanderen	0.93	0.74		1.16
	Vlaams Brabant	1.04	0.82		1.33

The degrees of freedom in computing the confidence limits is 13,812. Effects for categorical variables represent a comparison with the reference level. The first level of each variable is the reference level

*GALI* Global Activity Limitations Indicator

### Propensity score matching results

**Fig. 3:**
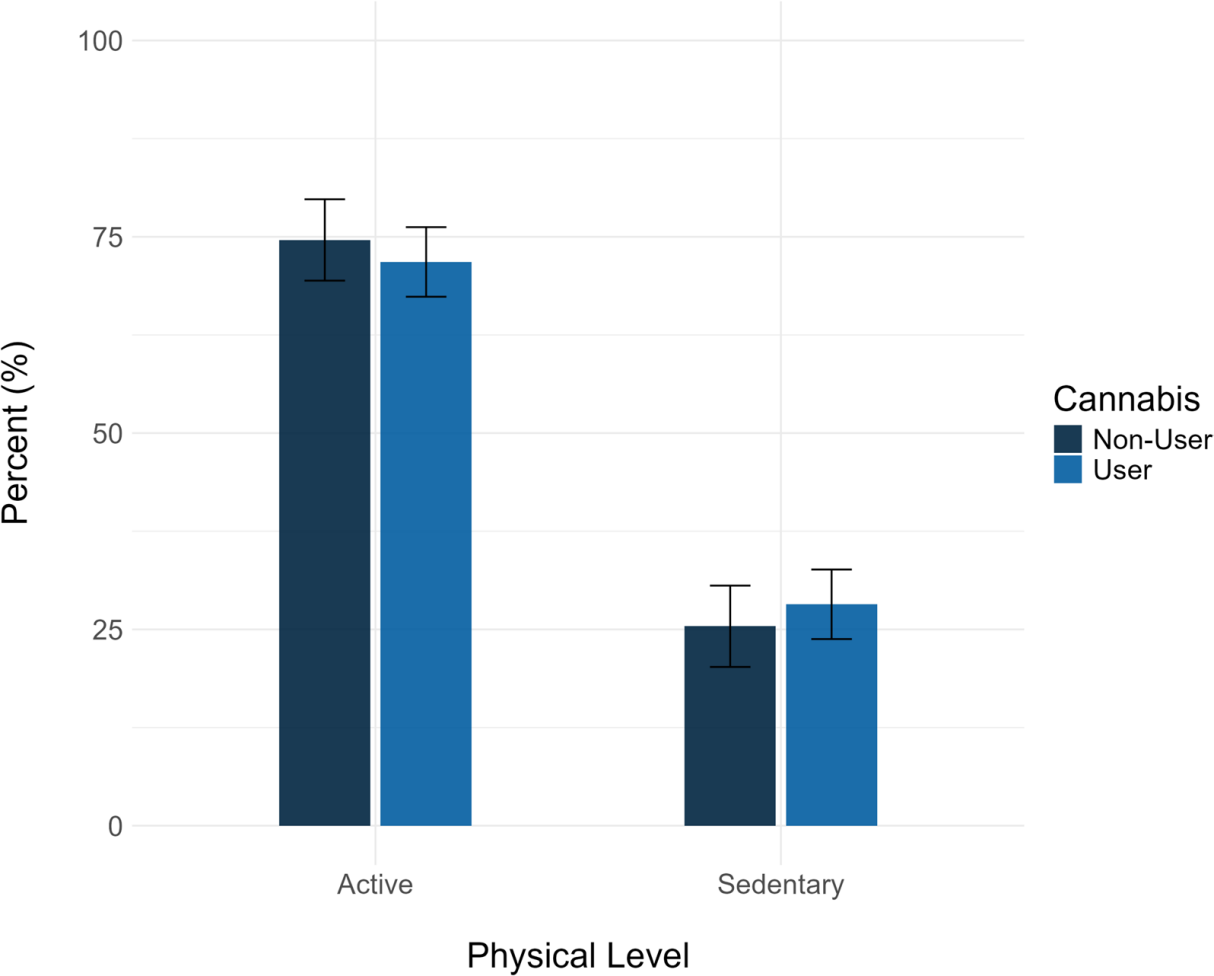
Estimated risks of active group membership for cannabis users and matched non-users. Legend: Error bars represent 95% confidence intervals. As the current estimates resulted from a propensity-matching analysis in which non-users were matched to users, the estimates reflect estimated percentages of active group membership in cannabis users and cannabis users, had they not used cannabis

The estimated frequency of active individuals in the user group was 71.8% (95% CI = [66.5; 76.9]), whereas the estimated frequency of active individuals in the matched non-user group was 74.6% (95% CI = [70.2; 79.1]), suggesting that users, had they not used cannabis in the last 30 days, would have a slightly greater probability of being in the active group. The resulting Risk Ratio (RR) of active group membership for users compared to users, had they not been using, was estimated to be 0.90 (95% CI = [0.70; 1.16]), suggesting that past-month cannabis use does not affect the risk of being in the active group (Fig. 3).

### Sensitivity analysis

All additional analyses resulted in minor quantitative, rather than qualitative changes. The same conclusion that past-month cannabis users and non-users show similar levels of leisure-time physical activity was reached in all additional analyses (see Supplementary Material 2).

## Discussion

The current study examined whether past-month cannabis use has a positive effect on the average amount of leisure time physical activity in a large and representative sample of the Belgian population. Additionally, our descriptive analyses provide insights into the defining characteristics of both the current users and non-users. Two complementary analyses revealed no evidence of a positive relation between past-month cannabis use and leisure-time physical activity after adjusting for potential confounders and the complex survey design.

The observation of no effect of past-month cannabis use on physical activity challenges a set of previously discussed recent studies reporting potential benefits (YorkWilliams 2019; Gillman et. al 2015; Ong et. al 2021; Gibson et al. 2023; Korn et al. 2018). The current study therefore serves as a cautionary note against premature conclusions, and underscores the need for further investigation of this important association. Moreover, the absence of an effect in the present study suggests that current cannabis users are not necessarily at a disadvantage in terms of physical activity levels (but note that absence of evidence does not equal evidence of absence). Furthermore, the suggested lack of association between cannabis use and physical activity can also be informative for other factors previously linked to cannabis use (e.g. obesity and cannabis consumption; Fearby et al. 2022). Finally, the propensity score model revealed that the probability of reporting past-month cannabis use decreased with age, was higher for men than women, and was higher for people with a generalized anxiety disorder, in line with previous findings (Trost et al. 2002; Crippa et al. 2009; Hayatbakhsh et al. 2007).

Our sample also better covers the effects of cannabis as it is used in daily life, compared with studies focusing on unrealistically smaller doses or recruiting subjects with a likely positive attitude towards cannabis use. Moreover, the use of the HIS dataset enabled us to easily adjust for other important health-related variables. Finally, by combining information from different HIS editions, we were able to study a relatively large number of cannabis users (a group that is usually relatively small in single samples).

At the methodological level, in the context of regression adjustment, the use of propensity scores (Hernán and Robins 2020) adds several important advantages: firstly, it allows for evaluation of the similarity of users and non-users in baseline covariates, a task that would otherwise be virtually impossible given the dimensionality of the data. Secondly, matching on the propensity scores provided a simpler interpretation in terms of the effect of cannabis in the users, while preventing extrapolation when no similar users and non-users (in terms of their propensity to use cannabis) can be found. Finally, the use of propensity scores, together with baseline covariates has been shown to make tests of the causal null-hypothesis more robust against potential misspecification of the regression model, by still delivering valid results in that case so long as the propensity score model is correctly specified (Vansteelandt and Daniel 2014).

Besides our large and representative sample and informative analyses, there are some considerations to keep in mind when interpreting the current findings. Firstly, although we specifically selected the variable of past-month cannabis use instead of past-year or even lifetime-cannabis use in order to better target regular users, this variable does not perfectly capture this target group. One can imagine that some of the respondents reporting past-month use were in fact first time users and might have not used thereafter, or that some users might have provided false information regarding their use, due to the illicit nature of cannabis in Belgium (though measures were taken to limit this influence, e.g., by having participants provide this information anonymously in a sealed envelope). Furthermore, no information was available on how cannabis was consumed in relation with leisure-time physical activity (before, during or after exercise), as was an important aspect in previous studies (YorkWilliams 2019), nor were we able to account for frequency/intensity of cannabis use or the consumption of other drugs. Finally, the current dataset did not have information regarding the precise cannabinoids that were used in the past month.

Similarly, while the variable leisure time physical activity provides a proxy for an individual’s general level of physical exercise, the absence of an association with past-month cannabis use may also be due to this variable not adequately capturing the type, intensity, or amount of participants’ physical activity. Akin to the potential effect of social desirability in reporting past-month cannabis use, respondents may have overreported the amount of physical exercise they got on a weekly basis. Alternative variables in the HIS dataset were considered (e.g., the binary indicators of meeting the World Health Organization (WHO) recommendations on health enhancing physical exercise), but were not selected due to this information being recorded for our selected HIS editions.

Finally, even though we selected important control variables, the possibility remains that some potential confounders were not available or included in the models. Importantly, the cross-sectional nature of the study additionally complicates a valid confounding adjustment as it is difficult to disentangle causes from effects of cannabis use. Moreover, we note the possible bias resulting from a complete case analysis (i.e., omitting missing data, see Supplementary Material 3 for a discussion) and the added value of the more efficient (but more complex) method of multiple imputation (Li et al. 2015). Finally, though we conducted a sensitivity analysis, it remains hard to formally verify the adequacy of logistic regression models, especially with complex survey designs (as these designs often violate core assumptions of standard logistic regression).

It is equally important to evaluate our findings in light of both the time and location in which these data were collected. Data collection for the included HIS editions dates back to as early as 2001. Since then, societal attitudes and actual use of cannabis have potentially changed. A recent study investigating the attitudes towards medicinal cannabis use in the general population of Belgium, for example, observed that a large portion of the respondents would be open to trying medical cannabis should this be needed (Pav et al. 2024). These generally high levels of acceptance and use, specifically in the Flemish region, were also reflected in the relatively large and increasing number of positive cannabis samples examined by the doping control laboratory in Ghent (Van Eenoo and Delbeke 2003). Taken together with the increasing trends in average potency and consumption frequency (Gisle and Drieskens 2018), it seems reasonable to expect differences in the association between cannabis use and physical activity over time. While data from the 2023 HIS edition were not available yet, it would be an interesting follow-up to repeat a similar analysis with these most recent data.

Another important contrast with previous studies is that our sample targeted the Belgian population as opposed to the majority of studies on the current effect taking place in the United States of America. As opposed to Belgium, a large number of states have legalized recreational and/or medical cannabis use (Cheng et al. 2003). Consequently, cannabis users in studies conducted in these states may show important differences with the user population queried in Belgium, both in frequency and accurate reporting of cannabis consumption.

Considering that cannabis use in the current and previous studies was mainly considered for recreational purposes, an interesting outstanding question is whether the association between cannabis use and physical exercise depends on the motivation for consumption (i.e., recreational versus medical). In the current study, no distinction could be made between these two types of users. Potentially, cannabis use for medical purposes could show a stronger (albeit negative) association with physical exercise, considering the likely presence of other health-related issues in this population. Follow-up studies with more information on the type of cannabis use will be important to differentiate the effect of cannabis use and the effect of general health status.

Additionally, despite the lack of evidence that people who use cannabis are at a disadvantage regarding physical activity levels, we argue that it is still crucial for public health agencies to continue promoting adequate levels of physical exercise, especially in the population of cannabis users. Despite the fact that the effects of cannabis use on mental and physical health are not yet fully-understood, increased levels of physical activity could potentially offset some of the commonly suggested adverse effects (predominantly of THC), such as on respiratory health (Pinckard et al. 2019), cardiovascular disease (Duncan et al. 2021), or mental health (Smith and Merwin 2020).

Taken together, our findings highlight possible important directions for future research. Firstly, using variables that better capture the constructs of interest (e.g., better/objective measures of cannabis use or physical activity) could help to draw more firm conclusions on the proposed association. A worthwhile follow-up study in this regard would include data from fewer waves for a more sensitive measure of physical activity and/or cannabis use. Secondly, as the possibility of unmeasured confounders remains an issue for observational studies, it will be of interest to revisit the considered scientific question based on longitudinal data and to explore how other factors may contribute to this important association (e.g., the role of exercise enjoyment, sleep quality, bodily pain, or the presence of cannabis use disorder). Finally, future studies, both on the current topic and more generally, could benefit from the incorporation of propensity based methods as applied here.

## Conclusion

We examined the association between past-month cannabis use and leisure-time physical activity in the Belgian population. To this end, we conducted two complementary analyses (a regression analysis and a propensity score matching analysis) on the Belgian Health Interview Survey dataset (2001–2018), a large and representative sample of our target population. Both analyses did not support a positive (or negative) effect of past-month cannabis use on physical activity levels while controlling for potential confounders and the complex survey design. While we argue that the stereotypical image of cannabis users having more sedentary lifestyles should be critically reevaluated in light of our and other research, we still consider promoting sufficient levels of physical exercise to be of utmost importance, both in user and non-user populations.

## Supplementary Information

Additional file 1.

Additional file 2.

Additional file 3.

## Data Availability

The data that support the findings of this study are available from Sciensano upon request.

## Abbreviations

CBD: Cannabidiol
THC: Delta-9-tetrahydrocannabinol
BMI: Body Mass Index
CI: Confidence Interval
HIS: Health Interview Survey
GALI: Global Activity Limitation Indicator
OR: Odds Ratio
RR: Risk Ratio
WHO: World Health Organization
