# Correction: Cannabis use is not associated with altered levels of physical activity: evidence from the repeated cross‑sectional Belgian Health Interview Survey

**DOI:** 10.1186/s42238-025-00290-y

**Published:** 2025-06-03

**Authors:** Brent Vernaillen, Brecht Devleesschauwer, Stijn Vansteelandt, Lydia Gisle, Sabine Drieskens, Elena Damian

**Affiliations:** 1 Department of Epidemiology and Public Health, Sciensano, Brussels Belgium; 2 Department of Applied Mathematics, Computer Science and Statistics, Ghent University, Ghent, Belgium; 3 Department of Translational Physiology, Infectiology and Public Health, Ghent University, Merelbeke, Belgium; 4 Present Address: Department of Experimental Psychology, Ghent University, H. Dunantlaan 2, Ghent, 9000 Belgium

**Correction: J Cannabis Res 7, 22 (2025)**

10.1186/s42238-025-00278-8

Following the publication of the original article, the author reported the following errors:

- On page 2, “see also 11 for a review” *Should be “Gillman et. al 2015”*.
- The link *‘chrome-extension://efaidnbmnnnibpcajpcglclefindmkaj/‘* should be removed from “Ethics approval and consent to participate” section.

The original article (Vernaillen et al. 2025) has been corrected.
