# Supplementary material for: Cannabis use is not associated with altered levels of physical activity: evidence from the repeated cross-sectional Belgian Health Interview Survey
Source: J Cannabis Res. 2025 Apr 25;7:22. doi: 10.1186/s42238-025-00278-8 (PMC12023420; doi:10.1186/s42238-025-00278-8)
Supplement: Supplementary file 1 — Additional file 1. [file 42238_2025_278_MOESM1_ESM.pdf]

Additional File 1. Statistical Analyses

Pre-processing

All pre-processing and descriptive analyses were conducted in R (1). The complete dataset (N = 62.895) containing data at the individual level was initially accessed, but only the selected variables were retained. First, given that the questions related to use of illicit drugs for example were restricted to respondents with an age between 15 and 64 years, all observations that did not meet this criterion (N = 35.431) were removed. The remaining 27.464 observations were subsequently filtered to retain only observations with no missing data, thus allowing for a complete case analysis. The final dataset to be used for analysis contained 19.936 observations. In a last step of pre-processing, all variables were given informative names and labels for further analysis, the physical activity variable was transformed to contain two levels only (sedentary and light/intensive) and all categorical variables were explicitly recoded as factors.

Regression analysis

Model motivation

Data resulting from national health interview surveys usually require additional attention in finding the most appropriate analysis method. The used model must take into account all of the unique challenges and implications of a complex survey design, as compared to more controlled, randomized experimental settings (2,3). The current dataset was no different, and the analysis must therefore take into account the different aspects of the design. We now discuss one by one the most important aspects of the design together with the implications for the analysis and how our model accounted for these.

A first important aspect of the data was that many of the included variables were

categorical (discrete), as opposed to continuous. The dependent variable of interest in the current study, namely physical activity level, was a binary variable which took on the values 1 for subjects belonging to the light/intensive activity group and 0 for subjects belonging to the more sedentary group. A natural choice for modelling binary data as a function of a set of covariates is the logistic regression model.

A second aspect was the challenge of how to appropriately select relevant variables to answer the scientific question of interest while accounting for potential confounders. In randomized experiments to investigate treatment effects, confounding is usually controlled by design (4,5) as the randomization of subjects to either treatment or control does not depend on any of the subject-specific characteristics, leading to an approximate balance in both observed and unobserved covariates for both groups. Observational studies face a unique challenge in estimating treatment effects in light of potential confounding, as distributions of observed and unobserved variables are likely to be different among the treated and untreated individuals. It is therefore of utmost importance to adjust for potentially confounding variables for valid estimation of treatment effects (5). Failure to account for covariates that are associated with the outcome variable or main predictor variable (or both) can result in biassed parameter estimates, ultimately causing incorrect inference (omitted variable bias; 6-9). In complex survey data, there is usually an abundance of measured variables, leading to the challenge of identifying important predictors to include in the model.

As was already mentioned in the descriptive analysis, initial variable selection was based on the hypothesized predictive value of covariates for either the main outcome variable (physical activity) or the main predictor of interest (cannabis use). Our approach to the subsequent building of the final model was inspired by the general method of ‘double selection’ (also referred to as post- double selection; 10,11). In this general framework, important predictors are identified in three steps: in a first step, the main predictor variable of

interest is regressed on the set of candidate variables, and covariates are subsequently identified for inclusion based on a selection procedure (e.g. stepwise selection, LASSO regression, …). In the second step, the same model is fit to the outcome variable, after which the selected variables are once again retained. The final step involves fitting a regression model to the outcome variable that contains as predictors the union of selected predictors from the first two steps in addition to the main predictor variable of interest. Intuitively, this approach leads to a parsimonious model that minimizes bias that could occur from wrongly excluding important predictors (6). The procedure gives candidate variables 'two chances' so to speak to be included in the model, while also allowing unimportant predictors to be dropped. In the current study, we sought to keep model building to a minimum, hence instead of applying the framework of double selection to the entirety of the HIS dataset to identify potential covariates, we used a simplified version on the subset resulting from the initial selection based on the literature.

As discussed in the previous section, observational studies pose an additional challenge for causal inference about a specific treatment effect as the researcher has no control over the allocation of treatment in the study population. In randomized controlled studies, this is usually not a problem as the treatment allocation (in this case cannabis use) does not depend on prior characteristics of the subjects by design. In theory, the only difference between the treated and untreated would be the actual treatment they received, making them comparable. In observational studies, this is usually not the case, as treated and untreated individuals are likely to differ in terms of their baseline characteristics. It is evident that comparison of treated and untreated individuals in the setting of observational studies is only meaningful when both groups have a large enough overlap in their baseline covariates (5,7). While adjusting for covariates in regression can help overcome confounding, it is hard to evaluate if the treated and untreated groups have sufficient overlap in terms of their

baseline covariate distributions (especially if the number of important covariates is large as it is nearly impossible to investigate differences in multivariate distributions for both groups). One approach that allows researchers to evaluate overlap between treated and untreated individuals is the use of propensity scores (5,7).

Propensity scores, as the name suggests, are the underlying probabilities (i.e. propensities) of individual participants to belong to the treatment group (8,9). In the current study, the propensity score was the unobserved probability of answering 'yes' to the question of past-month cannabis use in the HIS dataset. The propensity score can be thought of a single-value summary of an individual's baseline covariates that can subsequently be used in a number of adjustment techniques (e.g. matching, weighting, regression adjustment; (12,15-18). One advantage of inspecting the distribution of estimated propensity scores per user group is that it allows for efficient judgements of whether the groups have a sufficient overlap in baseline covariates to allow for meaningful comparison. Additionally, Vansteelandt & Daniel (10) showed that propensity adjustment in logistic regression makes the test of the causal null-hypothesis more robust against misspecification of the outcome regression model, by still delivering valid estimates, so long as the propensity score model is correctly specified. Given these attractive properties of propensity scores, the current study also included estimated propensities to adjust for confounding in the final regression model.

In addition to the scale of the outcome variable (categorical) and the issue of variable selection and confounding, the complex sampling design of the HIS dataset also poses unique challenges for data-analysis. Both stratification and multistage sampling are important to take into account when analyzing survey data (2,11,12). The HIS dataset contained 12 unique strata with predetermined sample sizes (5 provinces in Flanders, 5 provinces in Wallonia + 1 stratum corresponding to East Belgium and finally the region Brussels as the last stratum; 22). In addition to information on strata, the HIS dataset also provided sampling weights for every

individual in the sample, with the weights denoting how many individuals in the target population each respondent in the sample represents (the precise formula for the weights is described in 22).

The nested cluster structure in the data obviously violated one of the key assumptions in classical regression analysis, namely the assumption of independently distributed responses. To account for correlated responses, two main approaches are usually identified, namely a model-based approach and a design-based approach (13,14). The model-based approach involves explicitly modelling the correlation structure between the responses by specifying random effects, leading to so-called generalized linear mixed effects models. The second set of methods, design-based methods, usually make adjustments to the variance estimators of parameter estimates so that clustering information is taken into account. One well known instance of design-based approaches is the sandwich estimator (which allows for variance estimation that is robust against misspecification of the covariance structure, given a correctly specified mean structure; 25). Another commonly used technique (and the one used in the current study) is variance estimation based on a Taylor Series (TS) linearization (discussed later in this paragraph; 24,26). Our motivation for using the TS approach over multilevel models was based on both computational, and theoretical considerations. Firstly, given that the interest of the current study is in the effect of level 1 covariates (i.e. characteristics of the individual subjects) rather than higher-level characteristics or the specific covariance structure of the data, there would be no added value in using multilevel models (13). Secondly, several studies pointed out that -especially in large sample sizes- both the model-based and design-based methods typically yield qualitatively similar results (15). Finally, the use of TS approximation of estimator variance is well documented and easily implemented in standard software (e.g., the surveylogistic procedure in SAS, 28).

As opposed to standard linear regression, where a closed form solution to the normal

equations exists to find the OLS estimates of regression parameters, the estimators of regression parameters in a logistic regression model are non-linear, resulting in the need to use iterative algorithms to find maximum likelihood estimates. In order to estimate the variance of these non-linear estimators, a first-order Taylor expansion of the non-linear estimator is calculated around the population parameter to yield a linear approximation of the estimator that can subsequently be used in variance estimation. Specifically for the current study, the TS linearization also incorporates important aspects of the sampling design when approximating the non-linear estimator (e.g. clustering, strata and weights).

Taking together all of the aforementioned aspects of the HIS dataset and keeping in mind our research question, the model that seemed most appropriate in this case is a survey-weighted logistic regression model, where we aimed to adjust for confounding and try to prevent extrapolation beyond the data at hand by including a set of baseline covariates into the model, in addition to the estimated propensity scores. The (limited) model building followed three phases, inspired by the double selection literature (16,6): first, the treatment variable (cannabis use) was regressed on the set of covariates and type III tests were used to identify important predictors at the .05 significance level. Second, the same approach was applied to the outcome variable, by regressing physical activity on the same set of covariates in addition to cannabis use. Finally, a final model was fit to the outcome variable with cannabis use and the estimated propensity score as covariates. Both models (i.e. the propensity model and the outcome model) were fit using the surveylogistic procedure in SAS (17), that uses sampling weights, information about strata and clustering to estimate the regression parameters and associated variance. Weights were used in the estimation of regression parameters by including them into the formula for the pseudolikelihood and subsequently during the iterative estimation of parameters, as well as in the Taylor linearization approach to estimating the variance.

Variables

The initial set of covariates consisted of the following variables: Cannabis, Physical, Education, Income, Age, Gender, Depression, Anxiety, Urbanization and the Global Activity Limitations Indicator (GALI). Additionally, the variables Year and Province were included in the models as fixed effects to account for potential heterogeneity in responses of different years and geographical locations.

The binary variable ‘Physical activity’ was created from the original item ‘Past 12 months leisure time physical activity’ asking participants the question “What describes best your leisure time activities in the past 12 months?”. The original question allowed for the following answers: 1) ‘Hard training and competitive sport more than once a week’, 2) ‘Jogging and other recreational sports or gardening, at least 4 hours per week’, 3) ‘Jogging and other recreational sports or gardening, at the most 4 hours per week’, 4) ‘Walking, bicycling or other light activities at least 4 hours a week’, 5) ‘Walking, bicycling or other light activities at the most 4 hours a week’, and 6) ‘Reading, watching TV or other sedentary activities’. From this variable, responses 1 and 2 were recoded as ‘Intensive training / sport ≥ 4 hours per week’, responses 3 to 5 were recoded as ‘Sport < 4 hours / light activities’, and response 6 was recoded as ‘Sedentary activities’. Finally, the former two categories were recoded as ‘light/intensive physical activities’, resulting in the binary variable ‘Physical activity’ that was used in subsequent analyses.

Education was not recorded at the individual level, but refers to the highest diploma within the household of an individual. Similarly, income refers to quintiles of household equivalized income. Furthermore, GALI refers to the presence or absence of long-term functional limitations in typical activities in daily life (18). Depression was included as a binary variable reflecting the presence of ‘any depression’. This variable was constructed from two other variables in the HIS (Major Depressive Syndrome and Other Depression, both

were based on the Patient Health Questionnaire PHQ-9 (19). The other measure of mental health, anxiety, was also included as a binary variable. This variable assessed the presence of a Generalized Anxiety Disorder (GAD) based on the GAD-7 questionnaire (20). Finally, Urbanization refers to the level of morphologic and functional urbanization, and consisted of the categories 1) ‘Big cities and dense agglomerations’, 2) ‘Suburban/banlieus’, 3) ‘Urbanized municipalities’, and 4) 'Rural’.

Model Building

In a first step, we used the rationale of double variable selection to potentially refine our set of candidate predictors. To this end we first fit a survey-weighted logistic regression model to the ‘treatment’ variable, cannabis use, and retained the significant predictors. The model was defined as follows:

$$\log\left(\frac{P(Cannabis_i = 1|\boldsymbol{X_i})}{1 - P(Cannabis_i = 1|\boldsymbol{X_i})}\right) = \beta_1 Year_i + \beta_2 Education_i + \beta_3 Income_i + \beta_4 Age_i + \beta_5 Gender_i + \beta_6 Depression_i + \beta_7 Anxiety_i + \beta_8 Urbanization_i + \beta_9 GALI_i + \beta_{10} Province_i \tag{1}$$

With

$P(Cannabis_i = 1|\boldsymbol{X_i})$ The conditional probability of past-month cannabis use of subject i

$\beta_1, \ldots, \beta_{10}$ The regression coefficients

The model assumed that individual participant’s responses were i.i.d Bernoulli random variables with conditional mean μ_i which is the ‘risk’ of observing past-month cannabis use, conditional on the set of specific covariate values. We assumed the logarithm of the odds of past-month cannabis use depended linearly on the covariates. To account for the survey design, information about which of the 12 strata each subject belonged to was also specified, together with clustering at the household level, and sampling weights at the individual level. We used households as the clustering variable, as information on the primary sampling units

(i.e. municipalities) was not available. We assumed that the use of households already captured most of the dependencies between responses, as it is the lowest form of clustering present in the study, which usually has the strongest intra-class correlation. To obtain the maximum likelihood estimates of the free model parameters, the Fisher scoring (iteratively reweighted least squares) algorithm was used to optimize the pseudo-likelihood function. Estimation of the variance of parameter estimates proceeded via TS linearization.

Inspection of Type III tests for the estimated effects revealed that all variables except for Education, Income, Depression and GALI significantly predicted past month cannabis use at the α = .05 significance level (see Table S1.1 and see Table S1.2 for estimated odds ratios and confidence intervals)

Table S1.1

Results of the cannabis regression model (Type III tests)

Variable	F-value	Numerator Df	Denominator Df	P-value
Year	4.93	4	13809	<0.001 ***
Education	1.48	4	13809	0.207
Income	1.95	4	13809	0.099
Age	273.77	1	13812	<0.001 ***
Gender	103.86	1	13812	<0.001 ***
Depression	2.30	1	13812	0.129
Anxiety	10.17	1	13812	0.001 **
Urbanization	3.71	3	13810	0.011 *
GALI	1.35	1	13812	0.245
Province	3.36	10	13803	<0.001 ***

Note. $p < .05$*, $p < .01$**, $p < .001$***. GALI = Global Activity Limitations Indicator

Table S1.2

Estimated odd ratios and 95% confidence intervals based on the cannabis regression model

Variable	Effect	Odds Ratio	95% Confidence Bounds	
			Lower	Upper
Year	2001	1.00		
	2004	1.19	0.80	1.76
	2008	1.60	1.08	2.37
	2013	0.99	0.67	1.48
	2018	1.99	1.37	2.89
Education	Higher Education	1.00		
	Higher Secondary	1.29	0.97	1.72
	Lower Secondary	1.21	0.82	1.79
	No Diploma	0.83	0.46	1.48
	Other	1.86	0.82	4.21
Income	First	1.00		
	Second	1.12	0.74	1.68
	Third	0.68	0.45	0.98
	Fourth	1.03	0.70	1.51
	Fifth	1.01	0.68	1.53
Age	Age	0.91	0.90	0.98
Gender	Woman	1.00		
	Man	3.35	2.66	4.23
Depression	Yes	1.00		
	No	0.73	0.49	1.10
Anxiety	Yes	1.00		
	No	0.48	0.30	0.75
Urbanization	Rural	1.00		
	Suburban	0.86	0.51	1.46
	Urbanized	0.61	0.37	1.00
	Big Cities	1.20	0.75	1.92
GALI	Not Limited	1.00		
	Limited	1.23	0.87	1.74
Province	West-Vlaanderen	1.00		
	Antwerpen	1.62	0.90	2.93
	Waals Brabant	2.58	1.23	5.42
	Brussel	2.67	1.52	4.71
	Hainaut	1.26	0.68	2.35
	Limburg	0.93	0.44	2.00
	Liege	1.84	0.96	3.54
	Luxembourg	1.16	0.58	2.33
	Namur	0.87	0.40	1.91
	Oost-Vlaanderen	2.17	1.17	4.03
	Vlaams Brabant	1.77	0.81	3.90

Note. The degrees of freedom in computing the confidence limits is 13812. Effects for categorical variables represent a comparison with the reference level (first level of every variable). GALI = Global Activity Limitations Indicator

Next, a second survey-weighted logistic regression model was fit, but this time using the variable, physical activity level, as the outcome and including the same covariates as were used in the treatment regression model.

$$\log\left(\frac{P(Active_i = 1|\boldsymbol{X_i})}{1 - P(Active_i = 1|\boldsymbol{X_i})}\right) = \beta_1 Year_i + \beta_2 Education_i + \beta_3 Income_i + \beta_4 Age_i + \beta_5 Gender_i + \beta_6 Depression_i + \beta_7 Anxiety_i + \beta_8 Urbanization_i + \beta_9 GALI_i + \beta_{10} Province_i \quad (2)$$

With

$P(Active_i = 1|\boldsymbol{X_i})$ The conditional probability of light/intensive PA of subject i

$\beta_1, \ldots, \beta_{10}$ The regression coefficients

Estimation and variance estimation proceeded identically to the previously fit model. Based on type III tests (Table S1.3, see Table S1.4 for estimated odds ratios and confidence intervals), all variables significantly predicted membership of the light/intensive physical activity group at the alpha = .05 significance level, except for cannabis use. Based on the results of this first step, we concluded that all variables that were identified based on subject-matter grounds are important predictors of either the physical activity level, cannabis use, or both, and should therefore be included in the final propensity score model and outcome regression model.

Table S1.3

First outcome regression model results (Type III tests)

Variable	F-value	Numerator Df	Denominator Df	P-value
Year	5.26	4	13809	<0.001***
Education	41.19	4	13809	<0.001***
Cannabis	0.04	1	13812	0.836
Income	2.41	4	13809	0.047*
Age	17.95	1	13812	<0.001***
Gender	41.83	1	13812	<0.001***
Depression	35.53	1	13812	<0.001***
Anxiety	4.03	1	13812	0.045*
Urbanization	6.03	3	13810	<0.001***
GALI	93.29	1	13812	<0.001***

Note. $p < .05$*, $p < .01$**, $p < .001$***. GALI = Global Activity Limitations Indicator

Table S1.4

Estimated odds ratios and 95% confidence intervals based on the first outcome regression model

Variable	Effect	Odds Ratio	95% Confidence Bounds	
			Lower	Upper
Year	2001	1.00		
	2004	0.75	0.65	0.86
	2008	0.81	0.71	0.94
	2013	0.81	0.69	0.94
	2018	0.90	0.79	1.03
Education	Higher Education	1.00		
	Higher Secondary	1.59	1.42	1.79
	Lower Secondary	1.95	1.70	2.24
	No Diploma	2.84	2.39	3.37
	Other	1.97	1.30	2.99
Income	First	1.00		
	Second	0.91	0.78	1.07
	Third	0.85	0.73	0.99
	Fourth	0.86	0.73	1.00
	Fifth	0.79	0.68	0.92
Age	Age	0.99	0.99	1.00
Cannabis	Yes	1.00		
	No	1.03	0.78	1.36
Gender	Woman	1.00		
	Man	0.76	0.69	0.82
Depression	Yes	1.00		
	No	0.59	0.50	0.70
Anxiety	Yes	1.00		
	No	0.82	0.68	1.00
Urbanization	Rural	1.00		
	Suburban	1.00	0.84	1.19
	Urbanized	0.97	0.82	1.14
	Big Cities	1.26	1.07	1.47
GALI	Not Limited	1.00		
	Limited	1.77	1.57	1.98
Province	West-Vlaanderen	1.00		
	Antwerpen	0.78	0.63	0.96
	Waals Brabant	1.25	0.94	1.66
	Brussel	1.20	0.99	1.46
	Hainaut	1.71	1.42	2.07
	Limburg	1.06	0.83	1.35
	Liege	1.47	1.19	1.81
	Luxembourg	1.33	1.05	1.67
	Namur	1.19	0.94	1.52
	Oost-Vlaanderen	0.93	0.74	1.16
	Vlaams Brabant	1.04	0.82	1.33

Note. The degrees of freedom in computing the confidence limits is 13812. Effects for categorical variables represent a comparison with the reference level (first level of every variable). GALI = Global Activity Limitations Indicator

In a second step, we first used the cannabis model (as the relevant predictors were used already) to estimate individual participants' underlying propensities for past-month cannabis use to be used in the subsequent outcome regression model. Inspection of the estimated propensities per cannabis group allowed us to evaluate the degree of overlap between users and non-users, and to gain better insight in the danger of making extrapolations in the current study. A density plot (Figure S1.1) reveals three key insights. First, the distributions are highly left- skewed, confirming that cannabis use was a relatively rare event. Second, as would be expected, cannabis users had greater estimated propensities for cannabis use on average, based on their covariates. Third, and most importantly, though the distributions for users and non-users were clearly distinct, there was a good amount of overlap between both user levels.

With the set of covariates identified, we proceeded by fitting a final survey-weighted regression model to the outcome variable, with cannabis use as a main predictor in addition to the propensity score. Once again, clustering at the household level was specified, as well as information on the different strata and sampling weights. Estimation and variance estimation were carried out via the Fisher scoring algorithm to optimize the pseudo-likelihood function and TS linearization once more.

$$\log\left(\frac{P(Active_i = 1|\boldsymbol{X_i})}{1 - P(Active_i = 1|\boldsymbol{X_i})}\right) = \beta_1 Cannabis_i + \beta_2 Propensity_i \quad (3)$$

With

$P(Active_i = 1|\boldsymbol{X_i})$ The conditional probability of light/intensive PA of subject i

β_1, β_2 The regression coefficients

Figure S1.1

Distribution of estimated propensity scores for users and non-users based on a survey-weighted logistic regression model

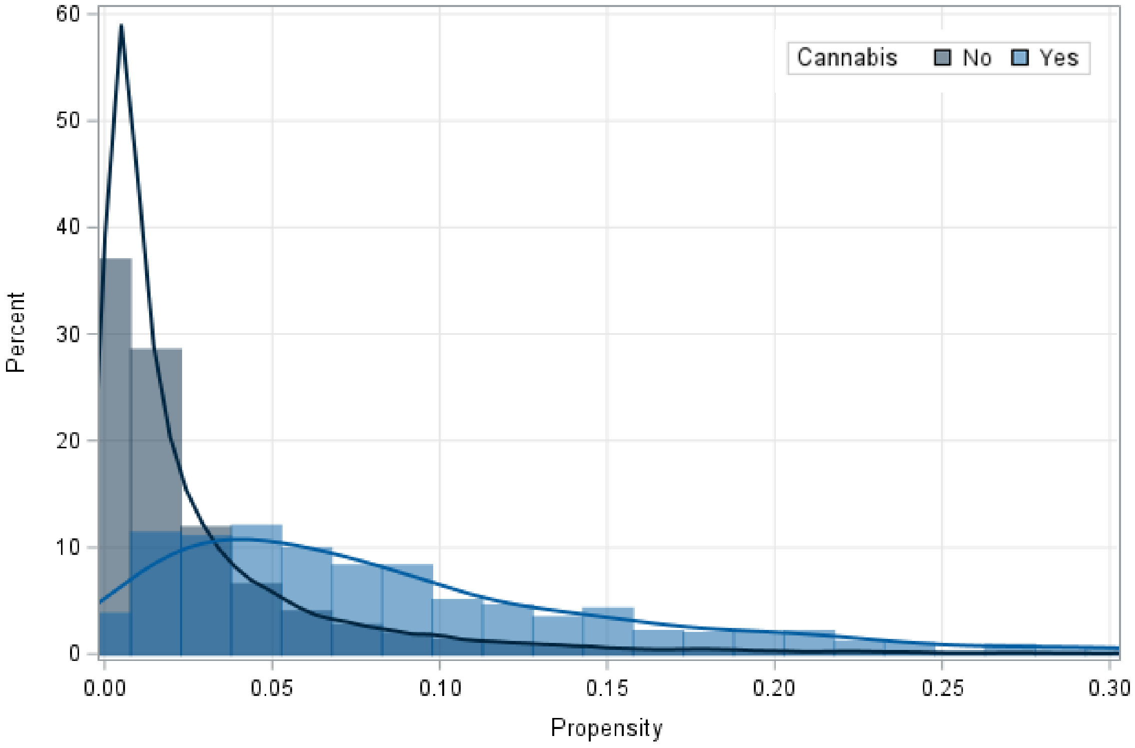

Note. Bars represent histograms of estimated propensity scores per group, whereas the smooth distributions represent estimated densities.

Propensity matching analysis

One potential drawback of the regression models above, especially with propensity score and covariate adjustment, is the rather complicated way of interpreting regression coefficients. The coefficients capture the natural logarithm of the estimated odds ratio of two levels of a covariate (e.g. user vs non-user in the categorical case). An alternative analysis that uses estimated propensity scores is propensity score matching. The general goal of this framework is to emulate the attractive property of balanced baseline covariates between treated and untreated individuals (usually present in randomized controlled trials), by matching the treatment groups on their estimated propensity scores. Using propensity scores

to match treated to untreated individuals allows researchers to estimate the average treatment effect in the treated, i.e., the observed effect (risk ratio or risk difference) if the treated individuals would have remained untreated. In the current study, this pertains to estimating the difference between the proportion of active individuals in the users and the proportion of active individuals in the users had they not used cannabis. The advantage of the current approach is that the results are easily interpretable and clearly convey the effect of interest.

Propensity score matching, i.e., finding the non-user for every user with the most similar estimated propensity for cannabis use, can be carried out in a lot of different ways. Different algorithms have been developed, with each one yielding slightly different results. According to a formal comparison based on simulations, Austin (21) found that nearest-neighbor caliper matching performed the best. In this algorithm, applied in the current study, users were selected one by one in a random fashion, each time defining an acceptable range on the logit of the propensity score of the non-users and selecting a random non-user that meets the criteria (if any). The use of an acceptable range around the estimated logit of each user is necessary as estimated propensities are typically quite unique due to rounding for example. The acceptable range is also commonly referred to as the caliper width as it allows researchers to flexibly place a constraint on how close the estimated logits of the non-users needs to be in order to be considered a valid candidate. The caliper width in this study was determined as 20% of the estimated standard deviation of the users' logit of the propensity score as shown to be optimal for estimating treatment effects in Austin (22). A suitable match could not be found for 4 out of the 618 users in the sample. The resulting propensity distributions for users and matched non-users are shown in Figure S1.2. As is evident from the figure, the distributions were almost identical, suggesting we established a good balance in baseline covariates between users and their matches.

Figure S1.2

Estimated propensities for users and matched non-users based on a survey-weighted logistic regression model

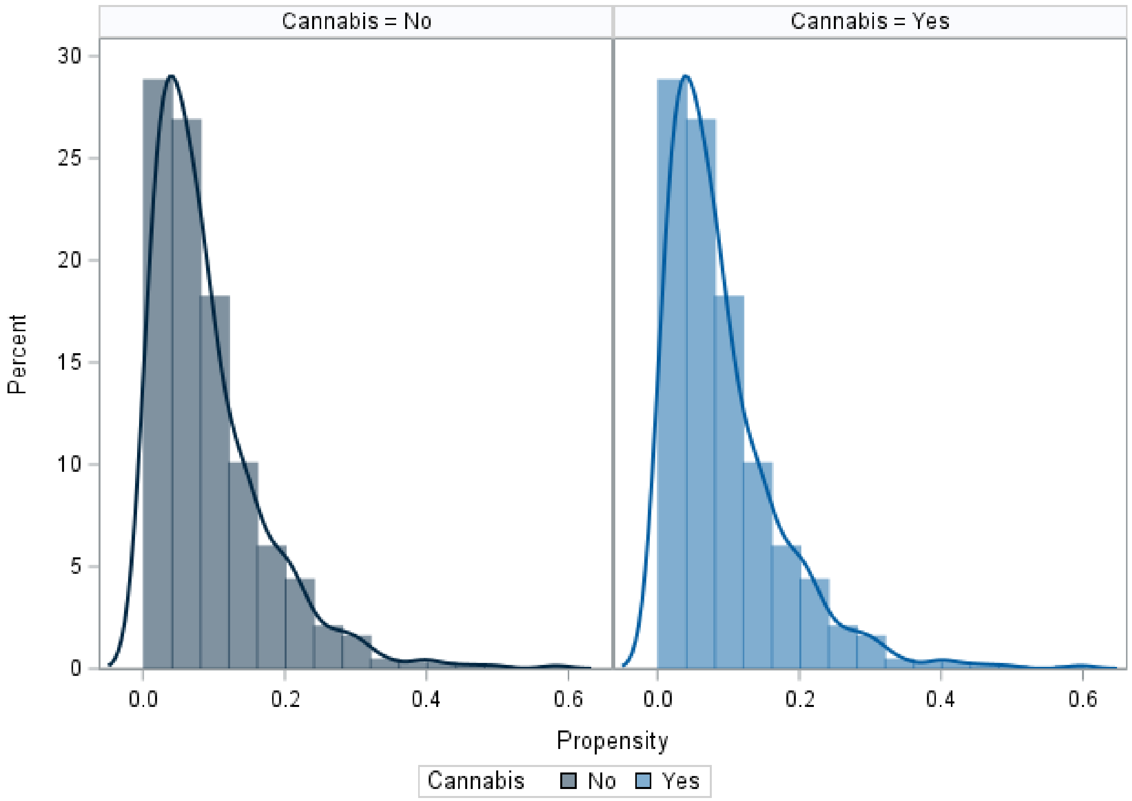

Note. Bars represent histograms of estimated propensity scores per group, whereas the smooth distributions represent estimated densities.

References

1. RStudio Team. RStudio: Integrated Development for R. RStudio, PBC, Boston, MA URL http://www.rstudio.com/. 2020.

2. Lumley T. Analysis of Complex Survey Samples. Journal of Statistical Software. 2004; 9(8).

3. Saylor J, Friedmann E, Lee HJ. Navigating Complex Sample Analysis Using National Survey Data. Nursing Research. 2012; 61(3): 231-237.

4. Jager KJ, Zoccali C, MacLeod AM, Dekker FW. Confounding: what it is and how to deal with it. Kidney International. 2008; 73(3): 256-260.

5. Hernán, M.A., Robins, J.M. Causal Inference: What If. Boca Raton: Chapman & Hall/CRC; 2020.

6. Urminsky O, Hansen C, Chernozhukov V. Using Double-Lasso Regression for Principled Variable Selection. SSRN Electronic Journal. 2016.

7. Kurth T, Walker AM, Glynn RJ, Chan KA, Gaziano JM, Berger K, et al. Results of Multivariable Logistic Regression, Propensity Matching, Propensity Adjustment, and Propensity-based Weighting under Conditions of Nonuniform Effect. American Journal of Epidemiology. 2006; 163(3): 262-270.

8. Rosenbaum PR, Rubin DB. Reducing bias in observational studies using subclassification on the propensity score. Journal of the American Statistical Association. 1984.

9. Rosenbaum PR, Rubin DB. The central role of the propensity score in observational studies for causal effects. Biometrika. 1983; 70(1): 41-55.

10. Vansteelandt S, Daniel RM. On regression adjustment for the propensity score. Statistics in Medicine. 2014; 33(23): 4053-4072.

11. Demarest S, Van der Heyden J, Charafeddine R, Drieskens S, Gisle L, Tafforeau J. Methodological basics and evolution of the Belgian health interview survey 1997–2008. Archives of Public Health. 2013; 71(1): 24.

12. Royal K. Survey research methods: A guide for creating post-stratification weights to correct for sample bias. Education in the Health Professions. 2019; 2(1): 48.

13. McNeish DM, Harring JR. Clustered data with small sample sizes: Comparing the performance of model-based and design-based approaches. Communications in Statistics - Simulation and Computation. 2017; 46(2): 855-869.

14. Huang FL. Alternatives to Multilevel Modeling for the Analysis of Clustered Data. The Journal of Experimental Education. 2016; 84(1): 175-196.

15. Gardiner JC, Luo Z, Roman LA. Fixed effects, random effects and GEE: What are the differences? Statistics in Medicine. 2009; 28(2): 221-239.

16. Belloni A, Chernozhukov V, Hansen C. Inference on Treatment Effects after Selection among High-Dimensional Controls. The Review of Economic Studies. 2014; 81(2): 608-650.

17. SAS Institute Inc. 2016. SAS/STAT® 14.2 User’s Guide. Cary, NC: SAS Institute Inc. [Online].

18. Van Oyen H, Van Der Heyden J, Perenboom R, Jagger C. Monitoring Population Disability: Evaluation of a New Global Activity Limitation Indicator (GALI). Sozial-und Praventivmedizin. 2006; 51(3): 153-161.

19. Spitzer R, Kroenke K, Williams J. Validation and utility of a self-report version of PRIME-MD: the PHQ primary care study. Primary Care Evaluation of Mental Disorders. Patient Health Questionnaire. JAMA. 1999 Nov; 282(18): 1737-44.

20. Spitzer R, Kroenke K, Williams J, Löwe B. A brief measure for assessing generalized anxiety disorder: the GAD-7. Arch Intern Med. 2006 May; 166(10): 1092-7.

21. Austin PC. A comparison of 12 algorithms for matching on the propensity score. Statistics in Medicine. 2014; 33(6): 1057-1069.

22. Austin PC. Some Methods of Propensity-Score Matching had Superior Performance to Others: Results of an Empirical Investigation and Monte Carlo simulations. Biometrical Journal. 2009; 51(1): 171-184.
