# Supplementary material for: Cannabis use is not associated with altered levels of physical activity: evidence from the repeated cross-sectional Belgian Health Interview Survey
Source: J Cannabis Res. 2025 Apr 25;7:22. doi: 10.1186/s42238-025-00278-8 (PMC12023420; doi:10.1186/s42238-025-00278-8)
Supplement: Supplementary file 2 — Additional file 2. [file 42238_2025_278_MOESM2_ESM.pdf]

ADDITIONAL FILE 2. SENSITIVITY ANALYSIS

A sensitivity analysis was performed to see if allowing for a more complex mean structure leads to qualitatively different results, both in the propensity score model and in the final outcome regression model. Two adaptations to the models were explored: adding all two-way interactions and adding a quadratic effect of the continuous age variable. This resulted in 4 additional analyses.

More complex propensity score models

Interestingly, literature suggests that specifying a more complex structure in models to estimate the propensity for subsequent regression adjustment leads to a smaller risk of overfitting compared to specifying a more complex structure for the outcome model (1). Additionally, including more predictors in the propensity model does not influence the interpretation of the final outcome regression model that uses a treatment variable and the estimated propensity as predictors. Regressing physical activity on cannabis use and the propensity score based on a propensity model with all two-way interactions, yielded approximately the same results, the estimated odds ratio for users was 0.95 (95%CI = [0.73, 1.25]), $t(13812) = -0.34$, $p = .731$ as opposed to the previous estimate of 0.97 (95%CI = [0.74, 1.28]), $t(13812) = -0.21$, $p = .836$. Adding the covariates in addition to the propensity score also did not induce qualitative changes. The model that included a quadratic effect of age also revealed no significant differences: the estimated odds ratio for users was 0.98 (95%CI = [0.74, 1.30]), $t(13812) = -0.14$, $p = .892$ as opposed to the previous estimate of 0.97 (95%CI = [0.74, 1.28]), $t(13812) = -0.21$, $p = .836$. The observation that richer models did not drastically change the estimates and inference do not suggest an important lack of fit.

More complex final regression models

The same adaptations were explored in the final outcome regression model, which again lead to the same conclusions. The observation that these extended models did not have a significant impact on parameter estimates and inference, shows that there is no evidence against the adequacy of the models presented in the previous sections.

Goodness-of-fit

Finally, as a more formal test of goodness-of-fit, the models presented in the main text were specified in Stata using the svy: logit command and subsequently subjected to a modified Hosmer–Lemeshow test that incorporates the complex survey design (2). The traditional Hosmer-Lemeshow test (3) is one of the most popular tests that provides insight into the general goodness-of-fit of a logistic regression model.

While this test is appropriate for simple random sampling designs (where responses can typically be assumed to be independent and identically distributed, i.i.d), the use of this test may not be valid with complex survey designs (2,4). To account for this, Archer & Lemeshow (2) developed a modified Hosmer- Lemeshow test that allows evaluating goodness-of-fit of a survey-weighted logistic regression. This modified statistic is calculated by first dividing the sample into deciles of risk based on the observed residuals (i.e. the first decile consists of subjects with the 10% lowest residuals. Second, a mean residual is estimated per subgroup by summing the decile's residuals, weighted by the corresponding sampling weights and dividing by the total of the sampling weights. Third, the variance of the estimated vector of mean residuals is estimated via Taylor series linearization. Finally, a Wald statistic is calculated from the estimated mean residuals and their estimated variances and subsequently compared to a reference F-distribution (2).

We first evaluated model fit in the propensity score model in Stata by using the svylogisticgof command. The modified Hosmer-Lemeshow test was found to be significant at the .05 significance level, indicating a lack of fit ($F(9,13804) = 2.85$, $p = .002$). Further

investigation of the model revealed that including the continuous variable age was responsible for the observed lack of fit. Moreover, adding an additional interaction term between age and education for example produced a much better fit ($F(9,13804) = 1.12$, $p = .347$). Running the same regression analysis using estimated propensities based on a model that included additional interactions between the covariates and age led to the same conclusions as before.

Second, we evaluated model fit in the same way for the final outcome regression model including the propensity score and all covariates. The modified Hosmer-Lemeshow test was not statistically significant at the $\alpha = .05$ level ($F(9,13804) = 1.35$, $p = .207$), suggesting no lack of fit. Though the propensity score model without any interaction terms was found to have a problematic fit to the data, inclusion of an interaction term between age and education remedied this problem. Additionally, our previously presented sensitivity analysis revealed that both a propensity model with and without interactions yielded similar results. We therefore decided to continue working with the models as presented in the main text, and concluded insufficient evidence for lack of fit. Note that, due to the lack of guidelines on the matter and the large dataset, we did not evaluate potentially influential observations.

REFERENCES

1.Millimet DL,&TR. On the specification of propensity scores, with applications to the analysis of trade policies. Journal of Business & Economic Statistics. 2009; 27(3): 397-415.

2. Archer KJ, Lemeshow S. Goodness-of-fit Test for a Logistic Regression Model Fitted using Survey Sample Data. The Stata Journal. 2006; 6(1): 97-105.

3. Hosmer DW, Lemesbow S. Goodness of fit tests for the multiple logistic regression model. Communications in statistics-Theory and Methods. 1980; 9(10): 1043-1069.

4. Archer KJ, Lemeshow S, Hosmer DW. Goodness-of-fit tests for logistic regression models when data are collected using a complex sampling design. Computational Statistics & Data Analysis. 2007; 51(9): 4450-4464.
