# Supplementary material for: Cannabis use is not associated with altered levels of physical activity: evidence from the repeated cross-sectional Belgian Health Interview Survey
Source: J Cannabis Res. 2025 Apr 25;7:22. doi: 10.1186/s42238-025-00278-8 (PMC12023420; doi:10.1186/s42238-025-00278-8)
Supplement: Supplementary file 3 — Additional file 3. [file 42238_2025_278_MOESM3_ESM.pdf]

ADDITIONAL FILE 3. A NOTE ON MISSING DATA

Missing responses to one or more items in a questionnaire are ubiquitous in survey research and can occur due to a number of reasons. Subjects can skip certain questions or forget to answer, but can also refuse to answer or provide inaccurate information. In the current study, we reported a complete case analyses (also referred to as listwise deletion) as we removed all observations that had an unobserved response in any of the selected variables. This is the most straightforward (though not the most efficient) way of dealing with missing data. A drawback of this approach is that it ignores the unobserved subjects altogether, and hence loses quite a lot of information. This loss of information ultimately leads to more uncertainty in model estimates, reflected in greater estimated variances. One could argue that, in this specific case, the dataset is sufficiently large and therefore sufficiently powered to detect a potential effect.

An additional danger of a complete case analysis is that it might induce bias in the parameter estimates. It could very well be the case that subjects with missingness in any of the variables (and hence excluded) differ in an important way with respect to the outcome variable of interest or any of the predictor variables. Ignoring these cases altogether in that case induces bias, as the sample is no longer representative of the population so that results cannot simply be generalized. In the current study, the complete case analysis amounts to assuming that the expected outcome, conditional on the covariates, is the same for the complete and incomplete cases. More formally, we say that $\mathbb{E}(Y \mid X, R = 1) = \mathbb{E}(Y \mid X)$, with Y the outcome, X the vector of covariates that were observed for a given missingness pattern and R the vector of missingness indicators. Though assumptions about missing data are typically impossible to verify due to the need to check for dependence on an unobserved quantity, if they hold, they guarantee valid parameter estimates and inference.

In the current dataset, missing values occurred for all variables except for year, age, gender, urbanization and province. There were 68 different patterns of missing variables in the dataset (see Table S3). Looking at the first two rows of Table S3, there were 19936 subjects with all variables observed and 3189 subjects with an unobserved income. Table S3 reveals that 582 participants did not report their past-month cannabis use. Due to the illicit nature of cannabis use, it might be the case that users are more likely to not report their cannabis use as compared to non-users. Finally, 1610 participants did not report their physical activity level. As physical activity is the outcome variable, we do not expect participants with missing data in any of the predictor variables to have a different distribution of physical exercise compared to complete cases. The reason for this assumed independence is that we do not see any reasons why participants with specific higher or lower values in certain covariates would be more/less likely to report their physical activity (e.g. why people with higher education would be more reluctant to report their physical activity level). Of course, this is still an assumption based on intuition and subject matter knowledge, there still might be a level of dependence between missingness in physical activity and any of the predictors.

Table S3

Patterns of missing data with respective frequencies

Cannabis	Physical	Education	Income	Depression	Anxiety	GALI	Frequency
X	X	X	X	X	X	X	19936
X	X	X		X	X	X	3189
X		X	X	X	X	X	1610
X		X		X	X	X	277
	X	X	X	X	X	X	582
	X	X		X	X	X	116
		X	X	X	X	X	173
		X		X	X	X	35
X	X	X	X	X		X	34
X	X	X		X		X	6
X		X	X	X		X	12
X		X		X		X	1
	X	X	X	X		X	10
	X	X		X		X	1
		X	X	X		X	12
		X		X		X	2
X	X	X	X		X	X	10
X	X	X			X	X	1
X		X	X		X	X	6
X		X			X	X	1
	X	X	X		X	X	2
		X	X		X	X	2
X	X	X	X			X	197
X	X	X				X	28
X		X	X			X	65
X		X				X	12
	X	X	X			X	87
	X	X				X	20
		X	X			X	84
		X				X	16
X	X	X	X	X	X		323
X	X	X		X	X		47
X		X	X	X	X		39
X		X		X	X		3
	X	X	X	X	X		26
	X	X		X	X		2
		X	X	X	X		16
X	X	X	X	X			1
X		X	X	X			2
	X	X	X	X			1
		X	X	X			2
X	X	X	X		X		1
X	X	X			X		1

	X	X	X		X		2
	X	X			X		1
		X	X		X		1
X	X	X	X				25
X	X	X					4
X		X	X				4
X		X					1
	X	X	X				26
	X	X					4
		X	X				37
		X					14
X	X		X	X	X	X	240
X	X			X	X	X	57
X			X	X	X	X	18
X				X	X	X	6
	X		X	X	X	X	13
	X			X	X	X	2
			X	X	X	X	3
X	X		X			X	1
X	X					X	1
	X		X			X	2
	X					X	1
						X	2
X	X		X	X	X		7
			X				1

Note. Cells containing an ‘X’ refers to the variable being observed, whereas empty cells reflect that the corresponding variable was not observed.
